# First principles investigation of halide based Rb_2_NaGaZ_6_ (Z = Br, I) double perovskites for energy harvesting applications

**DOI:** 10.1039/d3ra05060b

**Published:** 2023-12-08

**Authors:** Huda A. Alburaih, Ghulam M. Mustafa, Pakeeza Aymen Nawaz, Sadaf Saba, N. A. Noor, Asif Mahmood, Ramesh Sharma

**Affiliations:** a Department of Physics, College of Science, Princess Nourah Bint Abdulrahman University P.O. Box 84428 Riyadh 11671 Saudi Arabia; b Department of Physics, Division of Science and Technology, University of Education Lahore Punjab 54770 Pakistan dr.ghulam.muhammad@ue.edu.pk; c Department of Physics, University of Lahore Lahore Pakistan; d Center of Excellence in Solid State Physics, University of the Punjab Lahore Pakistan; e Department of Physics, RIPHAH International University, Campus Lahore Pakistan; f Chemical Engineering Department, College of Engineering, King Saud University Riyadh Saudi Arabia; g Dept. of Applied Science, Feroze Gandhi Institute of Engineering and Technology Raebareli 229001 Uttarpradesh India

## Abstract

Extensive investigations have been conducted on the thermoelectric and optoelectronic characteristics of double perovskite compounds using the full potential linearized augmented plane wave (FP-LAPW) approach. Here we investigated Rb_2_NaGaZ_6_ (Z = Br, I) to explore its band structure, and electronic, optical and transport properties. Born's stability criteria have confirmed the mechanical stability of these compounds. Analysis of the elastic properties reveals their ductile nature, as indicated by a Poisson coefficient (*υ*) greater than 0.26 and a Pugh ratio exceeding 1.75 for Rb_2_NaGaZ_6_ (Z = Br, I). Computation of the bandgap values shows that both compositions possess a direct bandgap nature, with respective values of 2.90 eV and 1.25 eV. This suggests that substituting Br with I brings the band edges closer together, resulting in a decrease in the bandgap value. The optical properties are assessed based on the absorption coefficient, reflectivity, and dielectric constants. The thermoelectric properties, including thermal and electrical conductivities, power factor (PF), and figure of merit (*ZT*), are determined using the BoltzTrap code. The *ZT* values indicate that both compositions exhibit promising potential for various transportation applications.

## Abstract 2

Crystal structure, band structure, variation of real part of dielectric constant and figure of merit of K_2_YAg(Br/I)_6_ double perovskites.

## Graphical abstract

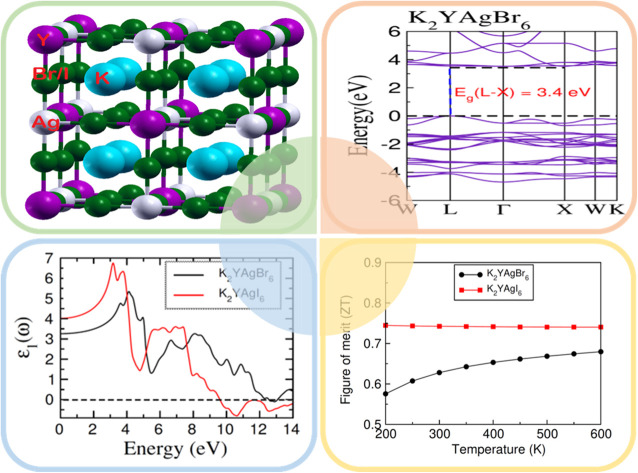

## 1. Introduction

In the era of constant technological evolution and expansion in economic advancement, to save time, effort, and energy for development we are dependent on machines, which results in environmental degradation.^1–4^ At the moment, to attain this energy demand, on a large scale, fossil fuels are being utilized which is consequently giving rise to environmental pollution and climate change. Therefore, to solve this issue renewable energy sources are required,^9,10^ like solar panels that use the photovoltaic effect to transform solar energy into electrical energy.^11,12^ In the beginning organic–inorganic lead-based perovskites solar cells were being used on a large scale as they can reach a great efficiency of 25.2%.^5,6^ But soon they experienced two crucial issues: one is the chemical instability and the other is the toxic nature of lead which harms the environment and public health and retards its feasible use.^7,8^ The extensive interest of researchers in double perovskites has grown over the last few years due to their conceivable implementation in renewable energy.^13,14^ By using these perovskites the ongoing record power conversion efficiency reaches up to 25.7%.^15^

For analyzing the most competent, reliable, and harmless substitutes, the double perovskites based on halide (X) (generally named as A_2_BB′X_6_) have been searched.^16–19^ Here A stands for alkali–alkaline earth metal and B and B′ stand for transition or post-transition metals which lead to new technological innovations.^20^ The double perovskites were introduced at the beginning of the 1950s.^20^ In the recent past, many studies have been reported after which, halide-based perovskites have achieved enormous attention, particularly for their applications in solar cells since they reveal direct band gaps, huge absorption coefficients in the UV range, and also big charge carrier mobility. The motive beyond the decision of choosing these particular compounds is their direct bandgap nature and utmost stable cubic structures.

In recent times, a lot of attention has been paid to exploring the potential of double perovskites for optoelectronic and thermoelectric devices. For instance, Rb_2_NaGaZ_6_ (Z = Br, I) which was studied by Behera and Mukherjee in 2022 which is a cubic double perovskite and bandgap calculation reveals the bandgap value of 1.81 eV for Rb_2_InBiCl_6_ and 1.32 eV for Rb_2_InBiBr_6_ with ductile nature. The compound Rb_2_InBiX_6_ (X = Cl, Br) have a high value of power factor which exhibit that these compounds will be potential candidates for use in thermoelectric (TE) devices.^21^ Similarly, Mebed *et al.* in 2022 reported Rb_2_AgBiX_6_ (X = Br, I) compound and noticed the bandgap value of 1.88 eV for Rb_2_AgBiX_6_ and 1.22 eV for Rb_2_AgBiI_6_. These compositions also exhibited notable TE features for TE generators.^22^ In addition, Mahmood *et al.* in 2021 submitted their work on Rb_2_TeX_6_ (X is replaced by Cl, Br, I) double perovskites that belong to space group *Fm*3*m* and possess a face-centered cubic structure. The bandgaps of these compounds were tuned from UV to visible region that is 3.2–1.80 eV as X is replaced by Cl, Br, and I which makes them suitable for solar-cell applications.^23^ In another report, Mahmood *et al.* in 2022 reported X_2_AgBiI_6_ (X will be changed by K, Rb, and Cs) and noted the bandgap values as 1.35, 1.26, and 1.30 eV for X = K, Cs, and Rb, respectively. These bandgap values with suitable *ZT* make them ideal for solar cell applications and thermoelectric generators.^25^ In addition to halide-based double perovskites, oxides-based double perovskites also captivated considerable attention like Sr_2_BTaO_6_ (B = Sb, Bi) was investigated by Manzoor *et al.* in 2022 and exhibited the indirect bandgap nature with bandgap values of 2.066 eV for Sr_2_SbTaO_6_ and 0.972 eV for Sr_2_BiTaO_6_ respectively. These materials are convenient for thermal devices and particularly Sr_2_BiTaO_6_ is more favorable for IR devices.^24^

This comprehensive literature exposed that the structural flexibility of double perovskites offers an extended bandgap tuning and allows them to fix themselves for advanced technological applications. Since there is still plenty of room at the bottom available to be explored to check the feasibility of double perovskites for optoelectronic applications. Thus, in the present communication, we analyzed the optoelectronic and thermoelectric properties of Rb_2_NaGa(Br/I)_6_ by utilizing the full-potential linearized augmented plane wave approach using DFT. So, the optical properties that are determined by utilizing TB-mBJ disclosed the direct bandgap nature. Optical parameters like refractive index *n*(*ω*), reflectivity *R*(*ω*), extinction coefficient *k*(*ω*), absorption coefficient *α*(*ω*), optical conductivity *σ*(*ω*), and dielectric constants are also computed.^26^ By using BoltzTrap code the temperature and chemical potential-dependent thermoelectric properties are estimated. Our current investigation is considered to provide new contestants for conceivable implementation in the later-development of renewable energy devices.

## 2. Computational details

This study investigates the unique characteristics of double perovskite compounds, specifically Rb_2_NaGaZ_6_ (Z = Br, I) focusing on their structural, optoelectronic, and thermoelectric properties. These compounds exhibited a cubic structure with the *Fm*3̄*m* space group. Our research employs the full-potential linearized augmented plane wave method (FP-LAPW) implemented through the WEIN2K code, which is based on Density Functional Theory (DFT) principles.^27,28^ The PBE-GGA approximation is utilized to determine the precise lattice constant of Rb_2_NaGaZ_6_ (Z = Br, I). For investigating the structural properties and determining the exchange co-relation potential (*V*_xc_), we employ the local density approximation (LDA) and the generalized gradient approximation (GGA). To accurately assess the bandgaps and band structures, we utilize the modified Becke Johnson (mBJ) approximation.^29,30^ In this method complete crystal is divided into two different categories related to the muffin tin, one is sphere and the other is an interstitial region, they are represented by the Fourier series. The input parameters for optimization and execution of SCF calculations are set as 1000 *k*-points of mesh size (12 × 12 × 12), angular momentum (*l*_Max_ = 10), Gaussian factor (*G*_Max_ = 16) and (*R*_MT_ × *K*_Max_) plane wave cutoff parameter = 8.

The thermoelectric properties are executed by the BoltzTrap code which is established on basis of Boltzmann Transport theory.^31^ Computation of thermoelectric characteristics including Seebeck coefficient (*S*), electrical (*σ*), and also thermal (*k*) conductivities by utilizing the following mathematical forms:^32^1
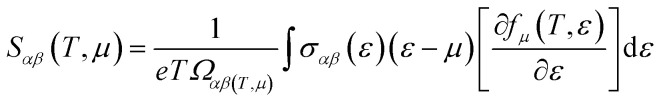
2
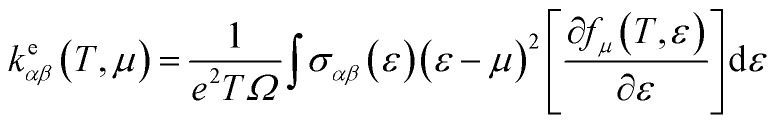
3
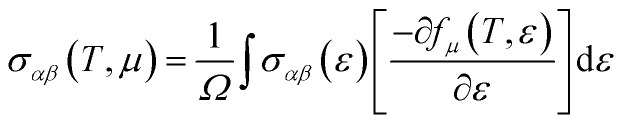


## 3. Result and discussion

### 3.1. Structural properties

**Fig. 1:**
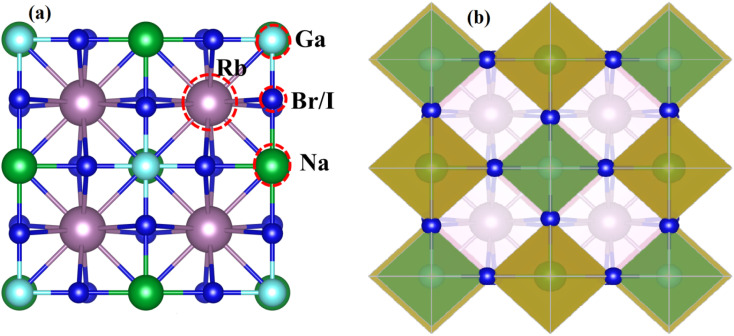
(a) Left side presents a cubic unit-cell ball and (b) the right side image shows polyhedral Rb_2_NaGaZ_6_ (Z = Br, I) double-perovskites.

**Fig. 2:**
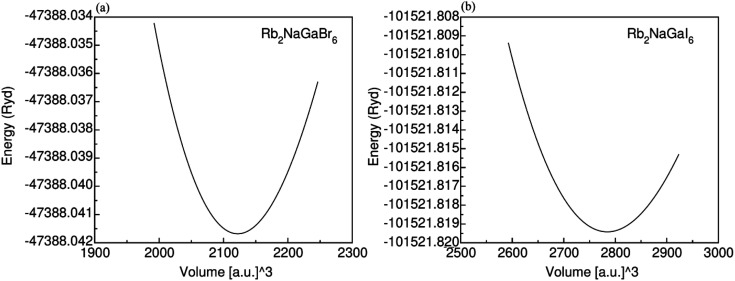
Energy volume optimization graph of (a) Rb_2_NaGaBr_6_, and (b) Rb_2_NaGaI_6_ double-perovskites.

Fig. 1 displays the cubic unit-cell of Rb_2_NaGaZ_6_ (Z = Br, I) double perovskites, with space group *Fm*3̄*m* [Fig. 1]. Constituent atoms Rb/Na/Ga/Z are occupied at coordinates (0.25, 0.25, 0.25)/(0.50, 0.50, 0.50)/(0, 0, 0)/(*x*, 0, 0) and Wyckoff positions at 4b/4a/8c and 24e.^33,34^ Volume optimization plot between volume and energy of both double perovskites Rb_2_NaGaBr_6_ and Rb_2_NaGaI_6_ investigated by the Brich–Murnaghan equation of state^35^ [Fig. 2].

The PBEsol-GGA functional is employed for the analysis of structural parameters. By comparing the ionic radii of iodine (I) and bromine (Br) [Table 2], it is observed that the larger ionic radius of iodine leads to an increase in the lattice constant (*a*_o_) from 10.79 Å to 11.81 Å. The calculated values of lattice parameters of Rb_2_NaGaBr_6_ are in good agreement with the values reported at materials project database.^36^ The bulk modulus (*B*_o_) indicates the material's ability to resist volume changes under applied pressure. The lattice constant, as well as Bulk modulus, are linked inversely thus as *a*_o_ is increasing so the *B*_o_ is assumed to decrease. Table 1 show a decrease in values of *B*_o_ from 24.3 GPa and 19.46 GPa as I is replaced with Br.

**Table 1:** Values of lattice constant *a*_o_ (Å), the bulk modulus *B*_o_ (GPa), enthalpy of formation Δ*H*_f_ (eV), the tolerance factor (*t*_G_) for cubic Rb_2_NaGaZ_6_ (Z = Br, I) double-perovskites

Parameters	Rb_2_NaGaBr_6_	Rb_2_NaGaI_6_
	PBEsol	PBEsol
*a*_o_	10.79	11.81
*B*_o_	24.34	19.46
*t*_G_	0.95	0.94
Δ*H*_f_	−1.53	−1.06
*C*_11_	39.71	50.8
*C*_12_	18.69	4.03
*C*_44_	10.62	8.91
*B*	25.60	19.52
*G*	10.57	13.20
*Y*	27.80	32.30
*B*/*G*	2.43	1.50
*υ*	0.32	0.22
*A*	1.01	0.38

To determine the stability of the crystal, Goldsmith tolerance factor *t*_G_ is computed. For a stable structure, its value must be between 0.81 and 1.11. Our calculated values of the Goldsmith tolerance factors for double perovskites Rb_2_NaGaI_6_ and Rb_2_NaGaBr_6_ are 0.94 and 0.95, respectively. A reduction in values of enthalpy of formation Δ*H*_f_ was observed from −1.53 to −1.06 eV as composition is shifted from Br to I.^37^ The negative sign express the discharge of energy during the construction of these compounds which confirms the stability of the compound. To inspect the mechanical behavior of compounds the elastic constants *C*_11_, *C*_22_, and *C*_44_ are computed.^38,39^ The Born Criterion condition shows as^40^

4 *C*_11_ − *C*_12_0, *C*_44_0, *C*_11_ + 2*C*_12_0, *B*_o_*C*_11_

They assist to find out the estimation of the stiffness of compounds against strains. To examine if the nature of compounds is ductile or brittle the value of the *B*/*G* Pugh ratio is measured. The Pugh value of Rb_2_NaGaBr_6_ (2.43) is higher than 1.75 and the value of the Poisson ratio (*υ*) *i.e.*, 0.32 is greater than 0.26 which reveals the ductile nature of this composition. However, when Br is replaced with I, the value of *B*/*G* and *υ* are reduced to 1.50 and 0.22 which uncovers the transformation of composition from ductile to brittle nature.

### 3.2. Electronic properties

**Fig. 3:**
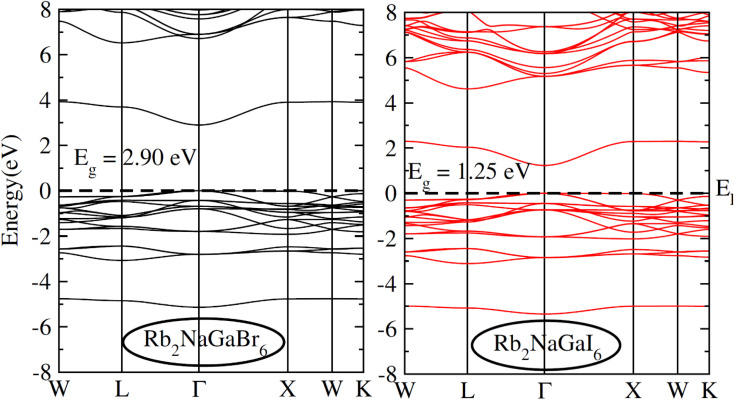
Representation of electronic band structures of Rb_2_NaGaZ_6_ (Z = Br, I).

To check the nature of the compound whether it is metallic, insulator or semiconductor we calculated the carrier concentration and their bandgap values. In Fig. 3 band structure of Rb_2_NaGaZ_6_ (Z = Br, I) is provided. Investigated band structures inclusive of high symmetry directions are described in 1st Brillouin zones and are computed by mBJ approximation. Fig. 3, depicted the direct bandgap semiconducting nature of compound Rb_2_NaGaZ_6_ (Z = Br, I) because the maxima of the valence band (VBM) and minima of the conduction band (CBM) lie at the same *Γ* point. The value of bandgap for Rb_2_NaGaBr_6_ is 2.90 eV and 1.25 eV for Rb_2_NaGaI_6_ noted from these band structures. It is clear that the band gap computed for Rb_2_NaGaBr_6_ using the TB-mBJ functionals gives larger value compared to the GGA values reported in materials project database where GGA functionals is employed.^36^ As I has higher ionic radii than Br, which lowered the conduction band edge and thus reduced the bandgap value for Rb_2_NaGaI_6_.

**Fig. 4:**
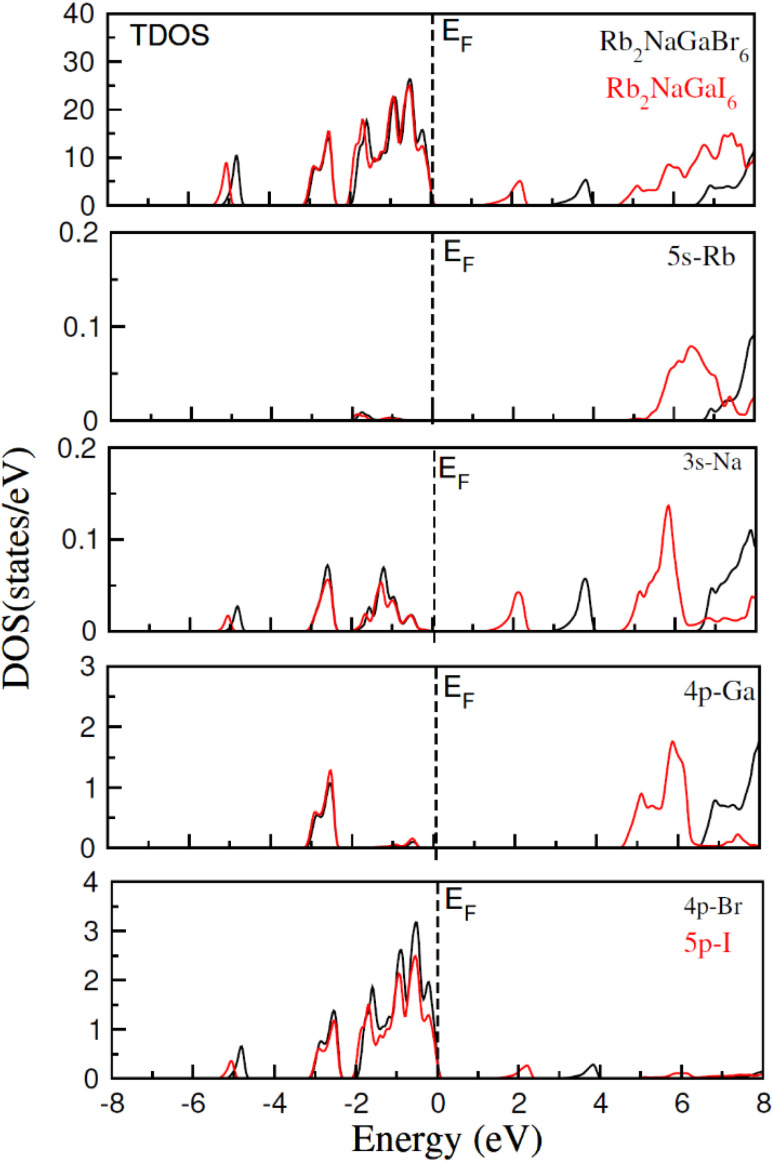
Show the total and partial density of states for Rb_2_NaGaZ_6_ (Z = Br, I).

In addition to this, the calculation of TDOS and PDOS was performed. The graphs depicting the density of states showcase the involvement of various states in the creation of energy bands, as indicated in Fig. 4. The TDOS graph reveals that the energy states in the valence band extend from −5.5 eV to the Fermi level. Furthermore, the conduction band edge is situated at 3.10 eV for Rb_2_NaGaBr_6_ and 1.45 eV for Rb_2_NaGaI_6_. Examining the PDOS graph closely, it becomes apparent that the valence and conduction band formation heavily relies on the 4p states of Rb and Ga, 3s states of Na, and 4p and 5p states of Br and I. The p-states of halogen atoms predominantly contribute to the valence band maxima, while the 3s-states of Na contribute to the conduction band minima. Interestingly, replacing Br with I causes the states in the conduction band to shift closer to the Fermi level, resulting in a reduced band gap value.

### 3.3. Optical properties

In order to assess the significance of Rb_2_NaGaZ_6_ double perovskites (where Z represents Br or I) in the context of solar cell usage, we conducted a thorough analysis of their optical characteristics. The effectiveness of capturing and utilizing incident light in any optoelectronic apparatus relies heavily on interband electronic transitions. A part of incident light induces polarization in material whereas the rest of the light is scattered. To quantify these features the most important parameter is *ε*(*ω*) dielectric constant which is utilized to optimize various parameters.

5 *ε* = *ε*_1_(*ω*) + î*ε*_2_(*ω*)

**Fig. 5:**
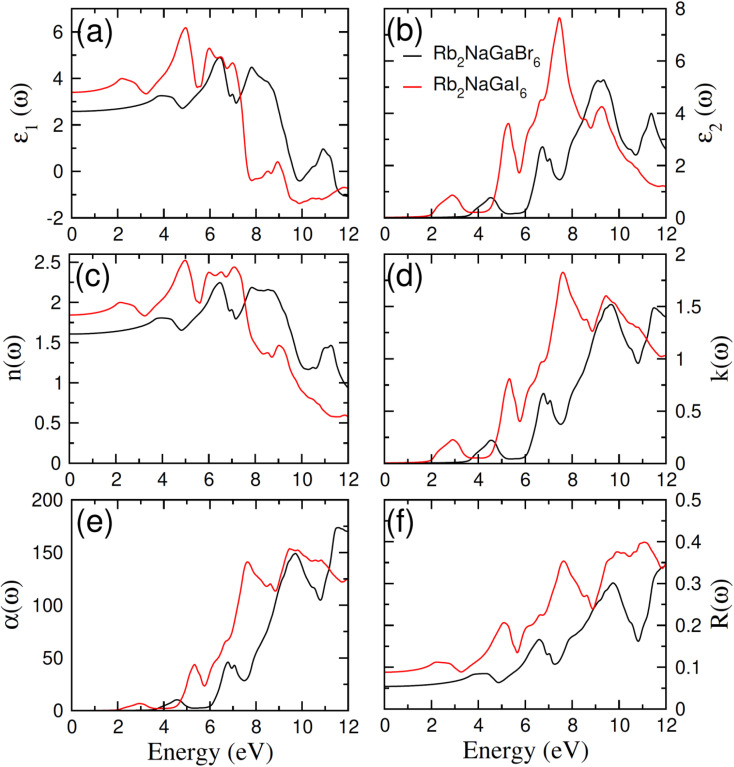
Characterization of (a) real and (b) imaginary values of the complex dielectric function, In (c) the refraction and (d) the extinction coefficient (e) absorption and (f) reflectivity for Rb_2_NaGaZ_6_ (Z = Br, I).

Here, *ε*_1_(*ω*) shows the dielectric constant's real part and represents the polarization capacity of material in contrast to the dispersion of impinging light which is determined by the imaginary part of the dielectric constant *ε*_2_(*ω*). The *ε*_1_(*ω*) and *ε*_2_(*ω*) are plotted for Rb_2_NaGaZ_6_ (Z = Br, I) and presented in Fig. 5(a) and (b). On zero energy, *ε*_1_(0) shows the value of 2.58 and 3.4 for Rb_2_NaGaBr_6_ and Rb_2_NaGaI_6_, respectively and that is called a static dielectric constant [see Table 2]. Static dielectric constant and bandgap are varies inversely. The real part of the dielectric constant is obtained by employing Kramers–Kroning relation:6
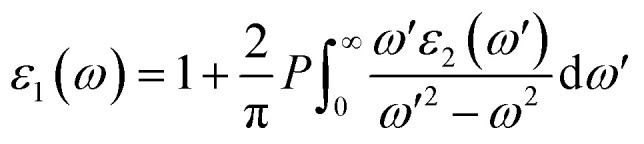
*P* is representing the principal integral.7
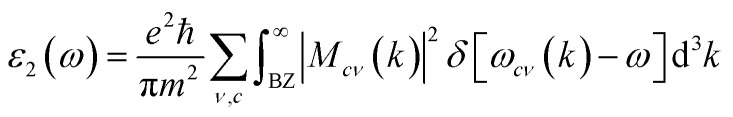


**Table 2:** Computed values of optical parameters at zero energy for Rb_2_NaGaZ_6_ (Z = Br, I)

Perovskite	*E*_g_ (eV)	*ε*_1_(0)	*n*(0)	*R*(0)
Rb_2_NaGaBr_6_	3.10	2.58	1.61	0.05
Rb_2_NaGaI_6_	1.45	3.4	1.84	0.08

The understanding of *ε*_1_(*ω*) and *ε*_2_(*ω*) let us to calculate refractive index *n*(*ω*), reflectivity *R*(*ω*), and absorption coefficient *α*(*ω*). These relations can be investigated by utilizing the following equations:8
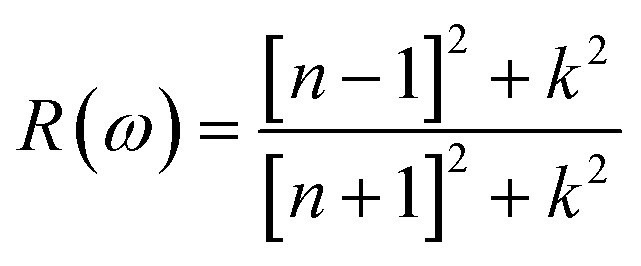
9
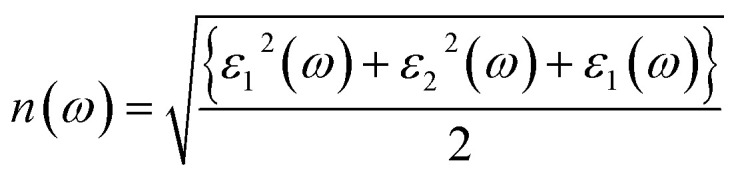


Incident energy lower than the bandgap is never able to generate electronic transitions from the valence band (VB) to the conduction band (CB). The value of total reflection varied from Br to I as 0.05 to 0.08 respectively on 0 energy. The refractive index as well as a real part of the dielectric constant are connected by:

10 *n*^2^ − *k*^2^ = *ε*_1_(*ω*)

Expand in the values of refractive index *n*(*ω*) is witnessed as 1.61 to 1.84 for Rb_2_NaGaBr_6_ and Rb_2_NaGaI_6_, respectively [see Table 2].11
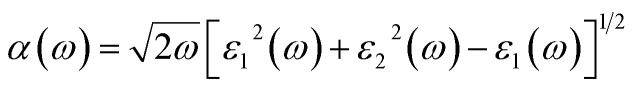


The coefficient of absorption evaluated the light energy that is absorbed by the semiconductor. The coefficient of extinction *k*(*ω*) informs us of the attenuation of light. The light energy attenuation is indicated by *α*(*ω*). The light energy that is captivated by the semiconductor is determined by the absorption coefficient *α*(*ω*). Additionally, *R*(*ω*) evaluated the reflection and scattering of light also Fig. 5. At zero energy. At zero energy *R*(0) for Rb_2_NaGaBr_6_ and Rb_2_NaGaI_6_ is 0.05 and 0.08 respectively represented in Table 2. The variation of absorption coefficient *α*(*ω*) with energy of the incident photons for Rb_2_NaGaI_6_ and Rb_2_NaGaBr_6_ are shown in Fig. 5(e). From this plot one can see that when the incident photon's energy is less than that of the band gap of the two materials, electromagnetic radiations are not absorbed. Based on the band gaps of these systems, Fig. 5(e) clearly shows the threshold in absorption for Rb_2_NaGaI_6_ is at lower energies compared to Rb_2_NaGaBr_6_. Since only for the case of optical band gap a value of *α*(*ω*) > 1.0 × 10^4^ cm^−1^ is realized,^41^Fig. 5(e) clearly shows photons absorbed by the studied materials are in accordance with the band gaps of Rb_2_NaGaI_6_ and Rb_2_NaGaBr_6_, indicating that these two materials are transparent to infrared photons. Compared to the case of hybrid perovskite materials, it can be seen that the optical absorption in the visible region for Rb_2_NaGaI_6_ is week.^42^ However, higher absorption of visible light is seen for Rb_2_NaGaI_6_ that can be assigned to the isolated conduction band at lower energies found for this material that causes absorption to begin at lower energies.

### 3.4. Thermal properties

**Fig. 6:**
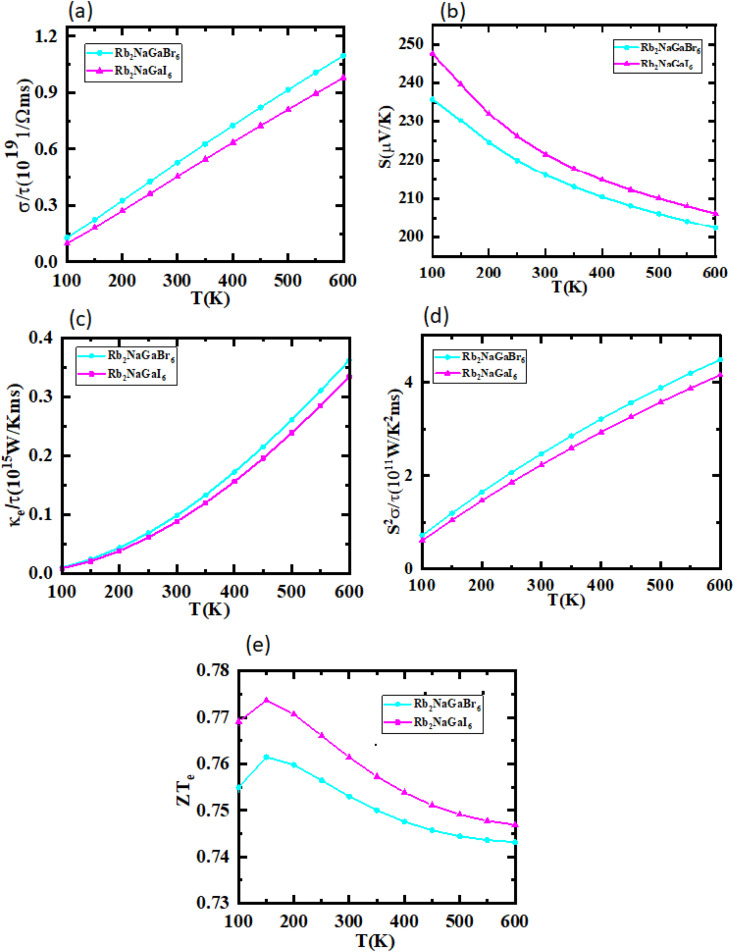
Representation of (a) electrical conductivity, (b) Seebeck coefficients, (c) thermal conductivity, (d) power factor, and (e) figure of merit plots against temperature for Rb_2_NaGaZ_6_ (Z = Br, I).

The utilization of certain materials capable of converting waste heat into electrical energy is highly suitable for thermoelectric (TE) devices, making them excellent candidates for energy harvesting. To determine the thermal properties of these double perovskites, calculations were performed using the BoltzTrap code, which is based on transport theory and implemented through WEIN2k. The resulting figures, shown in Fig. 6(a)–(e), illustrate the electrical and thermal conductivities (*σ*/*τ*) and (*κ*/*τ*), Seebeck coefficient (*S*), Power Factor (PF), and the figure of merit (*ZT*) across a temperature range of 200–600 K. A higher Seebeck coefficient indicates the favorable characteristics of a thermoelectric material. In order to achieve a greater *ZT* value, it is necessary for *S*, *σ*, and PF to be higher.

A good TE material essentially has bigger values of parameters besides thermal conductivity. The electrical conductivity of each composition approximates the flow of charge. To recognize the sort of material in case it is a conductor, insulator, or semi-conductor, the movement of charges is studied. For an outstanding TE device, the compound should essentially have a high estimation of (*σ*/*τ*). The estimation of the electrical conductivity reveals the potential of under-study compositions for designing new TE devices. Thermal conductivity map the flow of heat caused by the temperature gradient across the different ends of the material. This flow of heat could be either caused by electronic transportation called the electronic part of thermal conductivity (*κ*_e_) or lattice vibration called the lattice part of thermal conductivity (*κ*_l_). Rb_2_NaGaBr_6_ has a higher slope than Rb_2_NaGaI_6_ may be because electrons have the most energy. The determined smaller *k*_e_/*τ* and greater *σ*/*τ* is in the privilege of TE efficiency. Fermi level takes place at the *E*_g_ of the semiconductor also material application can be explained by free charge carrier which bounces from VB to CB. The Seebeck coefficient (*S*) which illustrates the fraction of potential difference induced due to temperature difference and suggested as thermo-power is indicated in Fig. 6.12
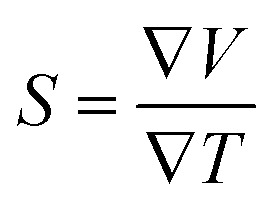


It can be either positive or negative depending on the nature of p-type and n-type.^43^ As the temperature is increased the *S* reduces. The figure of merit can be narrated by:13
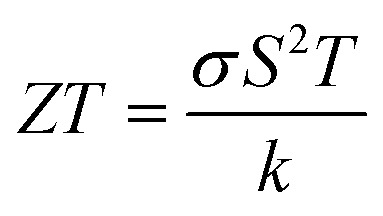


Generally, the TE performance of any composition is concluded by PF and *ZT*. The power factor measures the performance of the compound by a collaboration of *σ* and *S*. The escalation in PF at higher temperatures is examined as it may cause the existence of a greater number of charge carriers because of the large atomic number.14
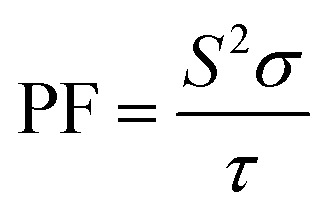


**Fig. 7:**
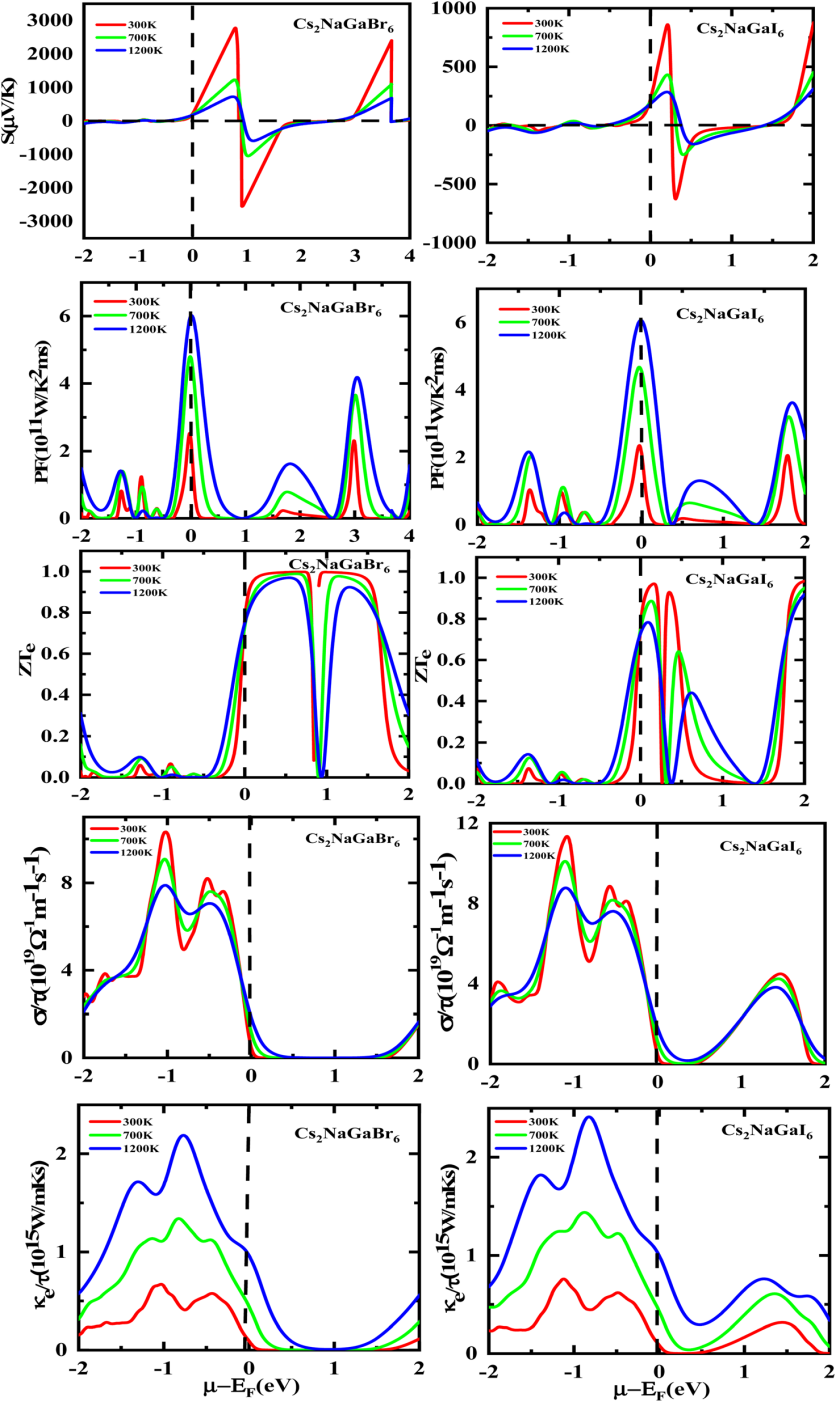
Rendering of Seebeck (*S*) coefficients, the power factor, the (*ZT*) figure of merit, electrical and thermal conductivity graphs contrary to the chemical potential for DPs Rb_2_NaGaBr_6_ and Rb_2_NaGaI_6_.

The semiconducting materials with an excess of electrons and holes are termed as n-type and p-type semiconductors, respectively. Although in chemical potential p-type is represented the negative value and n-type materials can be witnessed by a positive value. When we change the temperature, a change is produced in voltage which can be measured by the Seebeck coefficient. Which is written as 
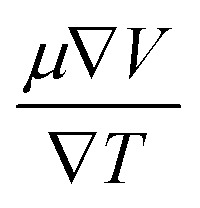
. In Fig. 7 we plot the graph of various thermoelectric parameters against chemical potential. The value of *S* ranges from +2700 μV K^−1^ to −2700 μV K^−1^ at 0.8 to 2 (*μ* − *E*_F_) (eV) and 0.3 to 0.4 (*μ* − *E*_F_) (eV) for Rb_2_NaGaBr_6_ and Rb_2_NaGaI_6_ respectively but as we increase the temperature from 300 K to 700 and 1200 K the curve decreases linearly. Though the negative side of chemical potential gives a minor curve and shows a straight line. The free motion of charge carriers results in the conduction of materials. Fig. 7 shows the values of *σ*/*τ* which reveals that as we increase the temperature from 100 K to 600 K the curve of electrical conductivities decreases. At 300 K temperature and −1.0 eV chemical potential, the value of *σ*/*τ* was noticed as 10.5 × 10^−19^ Ω^−1^ m^−1^ s^−1^ and at a maximum temperature of 600 K, the value of *σ*/*τ* is noticed on 4.8 × 10^−19^ Ω^−1^ m^−1^ s^−1^ and n-type is showing a minor contribution at 2 eV for Br. At 300 K Rb_2_NaGaI_6_ shows peak at 11.3 × 10^−19^ Ω^−1^ m^−1^ s^−1^ along with 0.8 eV and n-type doping taking part and demonstrates the curve at 4.2 × 10^−19^ Ω^−1^ m^−1^ s^−1^ along with 1.5 eV. There is a reverse connection between the Seebeck coefficient and electrical conductivity. The power factor graphs show an increasing trend as we increase the temperature for both compositions. At 300 K we obtain a peak at 2.3 × 10^11^ W K^−2^ m^−1^ s^−1^ along at 0 eV but as the temperature is increased up to 600 K a maximum curve is obtained at 6 × 10^11^ W K^−2^ m^−1^ s^−1^ and in p-type doping, the highest peak is obtained at 4.1 × 10^11^ W K^−2^ on 3.2 eV for Rb_2_NaGaBr_6_. For Rb_2_NaGaI_6_ in n-type doping, the maximum is noticed at 2.1 × 10^11^ W K^−2^, at 0 eV the power factor has a maximum curve at 6 × 10^11^ W K^−2^ and for p-type doping, the PF at 3.8 × 10^11^ W K^−2^ is obtained along 1.8 eV. We witnessed by stats that as we increase temperature we can obtain the maximum value of power factor. p-type doping has maximum contributions. Investigated value of the figure of merit investigated at 0.78 eV shows an increase but as we increase the temperature the curve decreases. It was found to be unitary at 300 K at 0.01 eV. The thermal conductivities for n-type and p-type doping is noticed as 0.65 × 10^15^ W m^−1^ K^−1^ s^−1^ and 0.80 × 10^15^ W m^−1^ K^−1^ s^−1^ at 300 K for Rb_2_NaGaZ_6_ (Z = Br, I) respectively.

**Fig. 8:**
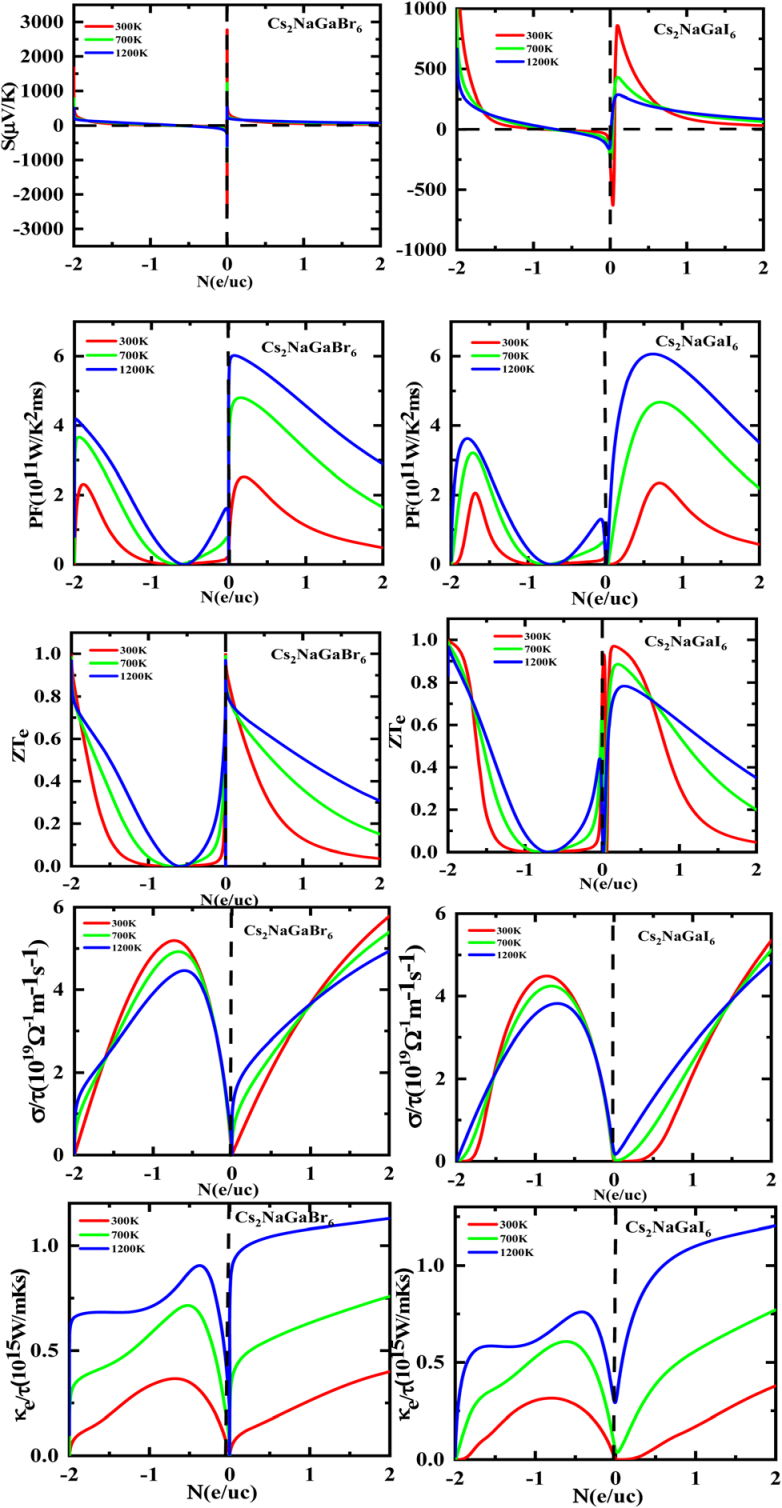
Images of Seebeck (*S*) coefficients, (PF) power factor, figure (*ZT*) of merit, electrical as well thermal conductivity graphs *versus* carrier concentration for DPs Cs_2_NaGaBr_6_ and Cs_2_NaGaI_6_.

Fig. 8 shows the carrier concentration plot. As the Seebeck coefficient and carrier concentration are related inversely, as we increase the concentration S decreases. At 250 (μV K^−1^) the concentration is at −1.7 (e per uc) and as we can see by the graph that by further increasing the value of carrier concentration the value of Seebeck is dropping. In p-type doping we witness a maximum peak for Rb_2_NaGaI_6_ at 850 (μV K^−1^) for 0.1 (e per uc) then it is decreasing linearly. The Power factor will give maximum value at a fixed point after that it will start declining. As PF depends upon *S* and *σ*. For Rb_2_NaGaI_6_ get maximum peak at 6.1 (μV K^−1^) for 0.6 (e per uc). The graph for the figure of merit depicts that as we increase the carrier concentration the *ZT* decreases. For Rb_2_NaGaBr_6_ we received a unitary value at 0.01 *ZT*_e_ and so for Rb_2_NaGaI_6_. Electrical and thermal conductivity has a direct relation with carrier concentration. As we start increasing value of Rb_2_NaGaBr_6_ for *σ*/*τ* from 0 N (e per uc) at 1.8 × 10^−19^ Ω^−1^ m^−1^ s^−1^ it starts increasing linearly. For Rb_2_NaGaI_6_ the curve increases from 0.2 × 10^−19^ Ω^−1^ m^−1^ s^−1^. For *k*_e_/*τ* as temperature goes on increasing from 300 K to 1200 K the concentration and thermal conductivity also increase. At 1.2510^15^ W m^−1^ K^−1^ s^−1^ along 2 N (e per uc) we get the maximum curve.

## 4. Conclusion

In nutshell, we investigated the Rb_2_NaGaZ_6_ (Z = Br, I) double perovskites to explore their potential for solar cell application. Opto-electronic and thermoelectric properties were computed by employing first-principles computation using DFT-based WEIN2k software. The structural stability of these compositions was calculated by measuring the value of enthalpy of formation which revealed that Rb_2_NaGaBr_6_ was more stable with Δ*H*_f_ = −1.53. Phase stability was assured by computed values of tolerance factor that was 0.95 and 0.94 for Br and I-based compositions. The analysis of band structure exhibited the direct bandgap nature of both of these compositions and a significant reduction in bandgap value from 2.90 to 1.25 eV upon replacement of Br with I. A low value of reflectivity enlarges the potential of our studied compounds. Electrical and thermal conductivities were greater for Rb_2_NaGaBr_6_. While Rb_2_NaGaI_6_ has a greater Seebeck coefficient. Better thermal efficiency with high absorption coefficient for UV photons make these compositions the best applicant for energy harvesting devices like solar cells.

## Data availability

The data included in this paper can be provided on request.

## Conflicts of interest

There is no conflicts to declare.

## Supplementary Material
